# Novel Strategy of Proxalutamide for the Treatment of Prostate Cancer through Coordinated Blockade of Lipogenesis and Androgen Receptor Axis

**DOI:** 10.3390/ijms222413222

**Published:** 2021-12-08

**Authors:** Yue Gu, Mengxia Xue, Qizhi Wang, Xiaodan Hong, Xinyu Wang, Fang Zhou, Jianguo Sun, Guangji Wang, Ying Peng

**Affiliations:** Key Laboratory of Drug Metabolism and Pharmacokinetics, State Key Laboratory of Natural Medicines, China Pharmaceutical University, 24 Tong Jia Xiang, Nanjing 210009, China; 15365017557@163.com (Y.G.); xue1342188683@163.com (M.X.); wangqqzhi@126.com (Q.W.); xiaodanhong0709@163.com (X.H.); wxyer@163.com (X.W.); zf1113@163.com (F.Z.); jgsun@cpu.edu.cn (J.S.); guangjiwang@hotmail.com (G.W.)

**Keywords:** proxalutamide, prostate cancer, androgen receptor, lipidomics, lipogenesis

## Abstract

**Objective:** Prostate cancer (PCa) is the most common malignant tumor diagnosed in men in developed countries. In developing countries, the PCa morbidity and mortality rates are also increasing rapidly. Since androgen receptor (AR) is a key driver and plays a critical role in the regulation of PCa development, AR-targeted agents provide a key component of current therapy regimens. However, even new-generation AR antagonists are prone to drug resistance, and there is currently no effective strategy for overcoming advanced PCa aggressiveness, including drug-resistance progression. The aim of this study was to evaluate the potential efficacy and novel therapy strategy of proxalutamide (a newly developed AR antagonist) in PCa. **Methods:** Four PCa cell lines with various biological heterogeneities were utilized in this study, namely, androgen-sensitive/-insensitive with/without AR expression. Proliferation, migration and apoptosis assays in PCa cells were used to evaluate the effective therapeutic activity of proxalutamide. The changes in lipid droplet accumulation and lipidomic profiles were analyzed to determine the influence of proxalutamide on lipogenesis in PCa cells. The molecular basis of the effects of proxalutamide on lipogenesis and the AR axis was then further investigated. **Results:** Proxalutamide significantly inhibited the proliferation and migration of PCa cells, and its inhibitory effect was superior to that of enzalutamide (Enz, second-generation AR antagonist). Proxalutamide induced the caspase-dependent apoptosis of PCa cells. Proxalutamide significantly diminished the level of lipid droplets in PCa cells, changed the lipid profile of PCa cells and reduced the content of most lipids (especially triglycerides) in PCa cells. Proxalutamide attenuated de novo lipogenesis by inhibiting the expression of ATP citrate lyase (ACL), acetyl CoA carboxylase (ACC), fatty acid synthase (FASN) and sterol regulatory element-binding protein-1 (SREBP-1). Moreover, proxalutamide also decreased AR expression in PCa cells, and its inhibitory effect on lipogenesis did not depend on its ability to down-regulate AR expression. However, Enz had no effect on AR expression, lipid accumulation or lipid de novo synthesis in PCa cells. **Conclusions:** By co-targeting the AR axis and endogenous adipogenesis, a novel and promising strategy was established for proxalutamide to combat the progress of PCa. The unique effect of proxalutamide on the metabolic reprogramming of PCa provides a potential solution to overcome the resistance of current AR-targeted therapy, which will help to effectively prolong its clinical service life.

## 1. Introduction

The prostate is a unique male organ and the largest accessory gland in the male reproductive system. Prostate disease is a common disease in adult men, usually including prostatitis [1], prostatic hyperplasia [2] and prostate cancer (PCa) [3]. Prostatitis mostly occurs in middle-aged and young men, and prostatic hyperplasia mostly occurs in middle-aged and old men; they are all curable. However, PCa is among the most common cancers in men and among the most common causes of cancer death in men. It is estimated that there are more than one million new cases worldwide each year [4,5]. The prostate is an androgen-dependent organ found exclusively in men, so most PCa tumors maintain this androgen dependence, at least initially. Consequently, androgen deprivation therapy (ADT) has always played a crucial role in the treatment of PCa, typically medical castration or surgical castration [6]. Responses to ADT are generally positive in the early stages of PCa, as reflected in reduced prostate-specific antigen (PSA) in the circulation, improved osteodynia, and the stabilization of tumor burden [7]. However, the response to ADT wanes over time, and the cancer invariably progresses to castration-resistant prostate cancer (CRPC) after a median time of 18–24 months of ADT [8]. CRPC accounts for the majority of PCa deaths. Therefore, CRPC has been the focus of basic research and drug development in recent years.

The proliferation and survival of PCa cells are dependent on signaling from the activated androgen receptor (AR) [9]. Although the exact pathophysiology of CRPC is not fully understood, it has been demonstrated that AR is still a cornerstone target as CRPC still relies on AR signaling [10]. The first-generation AR antagonists, flutamide and bicalutamide, were used earliest in the clinical treatment of CRPC patients. However, the positive effects of ADT on AR signaling are temporary for CRPC [11]. Elevated AR expression, or mutations in tumor cells, causing resistance to first-generation AR antagonists, is considered the main driver of CRPC [12]. For these AR mutants, the first-generation AR antagonists were demonstrated to have partial agonist effects, resulting in the further progression of PCa [13]. Although some patients with CRPC can continue to respond to second and third-generation AR antagonists, such as enzalutamide and apalutamide, the clinical outcome is still not satisfactory (short duration and insufficient activity) [14].

Here, we present proxalutamide, a novel generation of AR antagonists, designed by Suzhou Kintor Pharmaceutical, Inc. (Suzhou, Jiangsu, China). Proxalutamide significantly inhibited the growth of PCa in vitro and in vivo [15]. The cell proliferation of PCa cells and the tumor volume of xenograft mice were significantly suppressed in the proxalutamide treatment group. Proxalutamide demonstrated a stronger potency to inhibit the binding of androgen to the AR ligand binding domain than enzalutamide (3.5×), and a stronger potency to block the gene transcription function of AR than enzalutamide (2–5×) [16]. Moreover, proxalutamide was demonstrated to block the transcriptional activity of both wild-type AR and clinically relevant AR mutants, while maintaining full antagonism in CRPC cells. In addition, proxalutamide effectively reduced the AR protein levels in PCa cells and xenograft mouse tumors [15]. As AR up-regulation is still the main driver of CRPC resistance [17], the down-regulation of AR protein levels by proxalutamide may enable it to retain efficacy against CRPC that has developed resistance to current AR-blockage treatments. Proxalutamide is currently undergoing phase III clinical trials for CRPC treatment. The clinical trial numbers include CTR20182095, CTR20180849, etc. In addition to the antagonist effects on the AR signaling pathway, we also found the inhibitory effects of proxalutamide on glutamine metabolism, redox homeostasis and de novo pyrimidine synthesis in AR-positive PCa cells, resulting in an enhancement in the cellular sensitivity to proxalutamide [18].

The prostate exhibits a unique metabolism that changes during initial neoplasia to aggressive PCa and metastasis [19]. PCa relies heavily on the consumption of lipids rather than the citric acid cycle to produce energy. PCa cells often utilize lipids derived from androgens through AR expression [20]. However, these cells can also utilize de novo lipid synthesis to produce fatty acids in order to obtain energy. This shift to a lipid-producing phenotype is a key turning point in the progression of PCa. The de novo lipid producers have the ability to produce the key energetic molecules for growth without the regulation of androgens, which means that the disease has progressed to be unresponsive to androgen deprivation therapy, known as CRPC [21]. The activation of de novo lipogenesis in cancer cells is increasingly recognized as a hallmark of aggressive cancers, especially for metastatic CRPC [22,23,24]. The key enzymes involved in de novo lipogenesis, such as fatty acid synthase (FASN), acetyl CoA carboxylase (ACC) and ATP-citrate lyase (ACLY), are often overexpressed in PCa [25,26,27]. Moreover, FASN, ACC and ACLY are all clinically unfavorable PCa biomarkers, and their overexpression is confirmed to be positively correlated with low survival rate and disease recurrence [28,29,30]. Targeting de novo lipogenesis has become an attractive and potential therapeutic strategy in the management of PCa [31,32].

In our previous studies, we confirmed that the inhibitory effect of proxalutamide on the proliferation of PCa cells is significantly stronger than that of enzalutamide (2–3×) and bicalutamide (3–6×) [18]. In this study, we further evaluated the anti-PCa efficacy of proxalutamide and determined the novel molecular basis of proxalutamide in androgen-dependent and -independent PCa cells. Proxalutamide significantly induced cell apoptosis and suppressed the migration ability of PCa cells. Proxalutamide triggered programmed cell death via activation of the caspase-dependent and RIPK-dependent apoptotic pathways in PCa cells. In addition, proxalutamide significantly decreased messenger RNA (mRNA) and the protein expression of FASN, ACC, ACLY and SREBP-1 in PCa cells. SREBP-1 is a transcription factor of sterol regulatory element binding protein-1, which mainly controls the expression of genes related to adipogenesis and fatty acid/lipid homeostasis [33]. Through inhibiting the expression of key enzymes involved in de novo lipogenesis, proxalutamide down-regulated the content of intracellular lipids and the amounts of lipid droplets in PCa cells. Proxalutamide also impaired the expression of AR in AR-positive PCa cells. Our study found that proxalutamide can synergistically block the SREBP-1/FASN/lipogenesis and AR signal axis of PCa cells, which provides new insight into the underlying molecular basis of the superior therapeutic activity of proxalutamide in PCa.

## 2. Results

### 2.1. Proxalutamide Suppresses PCa Cell Growth and Migration

**Figure 1:**
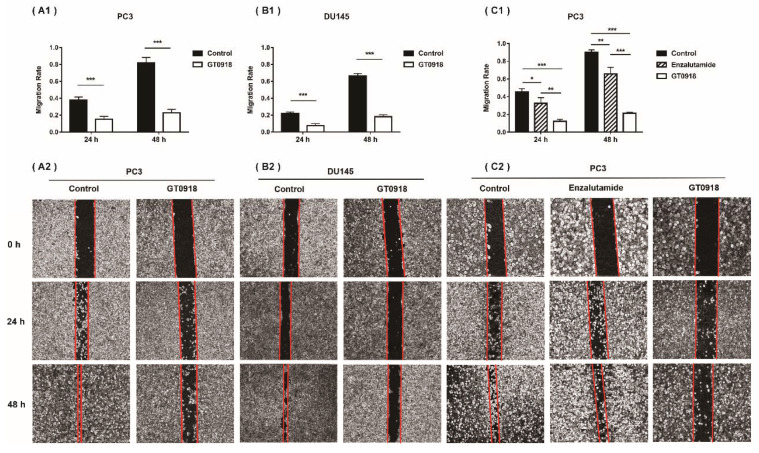
Proxalutamide (GT0918) inhibited the migration of PCa cells. (**A1**) Wound healing assay of PC3 cells after exposure to proxalutamide (40 μmol/L) or control vehicle. (**B1**) Wound healing assay of DU145 cells after exposure to proxalutamide (40 μmol/L) or control vehicle. (**C1**) Wound healing assay of PC3 cells after exposure to proxalutamide (40 μmol/L), enzalutamide (40 μmol/L) or control vehicle. Wound closure was analyzed by the migratory rate at 24 and 48 h, respectively. Migration rate was defined as the ratio of the difference between the wound area at 0 h and the test time after injury to the wound area at 0 h. Data represent the mean ± SD of three independent experiments. * *p* < 0.05, ** *p* < 0.01, *** *p* < 0.001. The typical images of the migration of PC3 and DU145 cells exposed to vehicle or tested drugs are shown in the bottom panel (such as **A2**, **B2** and **C2**).

According to our previous research [18], in both androgen-dependent LNCaP cells and castration-resistant 22RV1 cells, proxalutamide had superior inhibitory effects on PCa cell proliferation than bicalutamide and enzalutamide. The half inhibitory concentration (IC_50_) of proxalutamide in the growth of PCa cells is between 6.90 and 32.07 μmol/L. Then, the effects of proxalutamide on the PCa migratory potential—the hallmarks of progressive cancer cells—were investigated via a wound healing assay on two metastatic PCa cell lines (PC3 and DU145). As shown in Figure 1A,B, after 24 and 48 h of treatment, proxalutamide had a significant inhibitory effect on wound closure compared with the vehicle control group both in PC3 and DU145 cells. In addition, as shown in Figure 1C, the inhibitory effect of proxalutamide on PCa cell migration was significantly stronger than that of enzalutamide (the second-generation clinical AR antagonist with the same core structure as proxalutamide).

### 2.2. Proxalutamide Induces Caspase-Dependent Apoptosis Leading to PCa Cell Death

**Figure 2:**
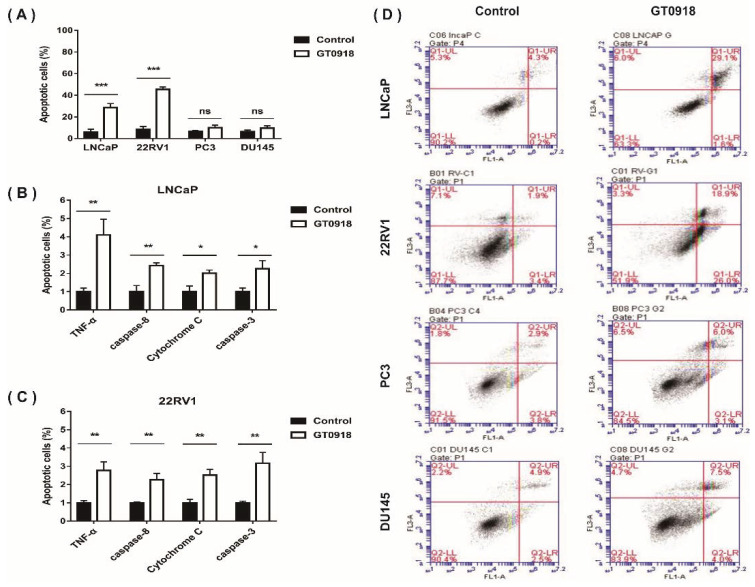
Proxalutamide (GT0918) induces caspase-dependent apoptosis, leading to PCa cell death. (**A**,**D**) Four PCa cell lines (LNCaP, 22RV1, PC3 and DU145) were exposed to proxalutamide or control vehicle for 48 h. (**A**) Apoptotic cells (%) were measured through Annexin V/PI staining flow cytometry assay. Data represent the mean ± SD of three independent samples, ‘ns’ *p* > 0.05, *** *p* < 0.001. (**D**) The typical images of flow cytometry assay in PCa cells. (**B**,**C**) The mRNA expression of *TNF*-α, *caspase*-8, *Cytochrome C* and *caspase*-3 after exposure to proxalutamide or control vehicle in LNCaP and 22RV1 cells, respectively. The relative mRNA level was defined as 1.0 (fold) in the vehicle-treated cells. Data were normalized by β-actin mRNA expression and are shown as the mean ± SD (*n* = 5), * *p* < 0.05, ** *p* < 0.01.

A tumor is considered to be a disease in which there is too little apoptosis and too much proliferation. If the proliferation of tumor cells can be inhibited and their apoptosis can be increased, the tumor may stop growing [34]. To explore whether proxalutamide leads to cell death, two AR-positive PCa cell lines (LNCaP and 22RV1) and two AR-negative Pca cell lines (PC3 and DU145) were selected to investigate the numbers (%) of apoptotic Pca cells before and after proxalutamide administration. As shown in Figure 2A, the percentage of apoptotic cells was 34.5 ± 3.8% in LNCaP and 38.8 ± 4.9% in 22RV1 cells treated with proxalutamide, respectively, while the percentages of vehicle-treated apoptotic Pca cells were only 5.7 ± 1.2% (LNCaP) and 12.9 ± 1.8% (22RV1). However, no significant influence of proxalutamide was found on the numbers of apoptotic cells of AR-negative Pca cells (PC3 and DU145). The results suggest that the cell apoptosis induced by proxalutamide may be correlated with the impairment of AR expression or AR signaling, as with other anti-prostate cancer drugs [35,36,37]. Apoptosis refers to cell death caused by the activation of caspase-dependent apoptotic responses through exogenous or endogenous pathways [38]. As shown in Figure 2C, proxalutamide significantly increased the mRNA levels of common apoptosis-related factors in LNCaP and 22RV1 cells, such as Tumor necrosis factor-alpha (*TNF*-α), *caspase*-8, *Cytochrome C* and *caspase*-3. The results suggest that proxalutamide promoted cell death via the induction of caspase-dependent apoptosis in AR-positive PCa cells.

### 2.3. Proxalutamide Reduces Lipid Enrichment in PCa Cells

**Figure 3:**
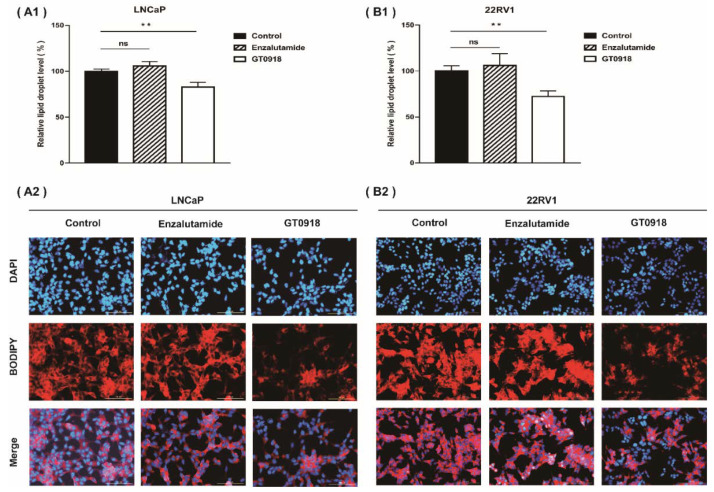
Proxalutamide (GT0918) reduced lipid droplet accumulation in LNCaP cells (**A**) and 22RV1 cells (**B**) examined by a BODIPY 558/568 C_12_ staining assay. Data of relative lipid droplet level (**A1**,**B1**) are shown as the mean ± SD of three independent samples, ‘ns’ *p* > 0.05, ** *p* < 0.01. The typical images of lipid droplet accumulation in PCa cells after exposure to proxalutamide (20 μmol/L), enzalutamide (20 μmol/L) or control vehicle are shown on (**A2**,**B2**). Scale bars = 100 μm.

According to our previous research [18], proxalutamide could enhance the sensitivity of PCa cells to the intervention of proxalutamide by affecting the endogenous metabolism of PCa cells. Abnormal lipid metabolism has long been recognized as a significant feature of the occurrence and development of PCa [39,40]. Even with ADT, the volume of fat and visceral fat increased significantly and continued to increase after treatment [41], leading to the relapse and further deterioration of the disease [42]. However, as shown in Figure 3, proxalutamide significantly diminished the levels of lipid droplets in LNCaP and 22RV1 cells, but enzalutamide did not have this effect. The results suggest that proxalutamide could combat lipid accumulation when used for ADT, which is beneficial for prolonging the progression-free period of the disease.

### 2.4. Proxalutamide Changes Lipid Profile of PCa Cells

**Figure 4:**
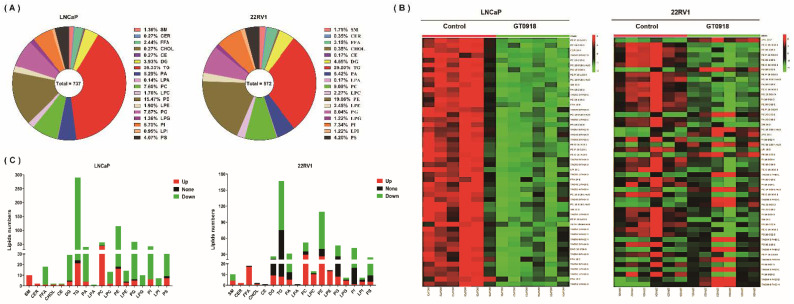
Lipidomic analysis to evaluate the effect of proxalutamide (GT0918) on PCa cells. (**A**) The total number and proportion of detected lipids of eighteen classes in LNCaP and 22RV1 cells through LC–MS/MS analysis. (**B**) Heat map of the intracellular content of the first 50 discriminant lipids with the largest difference between the treated and untreated cells. The test LNCaP and 22RV1 cells were treated with proxalutamide or control vehicle, respectively (*n* = 6). Red indicates a high content, and green indicates a low content. (**C**) The detailed number of lipids detected in each lipid category in LNCaP and 22RV1 cells, where red represents the number of lipids whose content was up-regulated after proxalutamide intervention, green represents down-regulated lipids and black represents lipids that did not change. SM: sphingomyelin; CER: ceramide; FFA: free fatty acids; CHOL: cholesterol; CE: cholesterol ester; DG: diglyceride; TG: triglyceride; PA: phosphatidic acid; LPA: lysophosphatidic acid; PC: phosphatidylcholine; LPC: lyso-phosphatidylcholine; PE: phosphatidylethanolamine; LPE: lyso-phosphatidylethanolamine; PG: phosphatidyl-glycerol; LPG: lyso-phosphatidylglycerol; PI: phosphatidylinositol; LPI: lyso-phosphatidylinositol; PS: phosphatidylserine.

Based on the importance of lipid metabolism in PCa, on the basis of the previous non-target metabolomics research [14], the effect of proxalutamide on the lipid profile of PCa cells was further studied by lipidomics analysis. As shown in Figure 4A, a total of 737 and 572 lipids in 18 lipid classes were detected in LNCaP and 22RV1 cells, respectively. Among them, approximately one third of the detected lipids were triglycerides (TGs). Through multivariate analysis, it was found that after the intervention of proxalutamide, most of the lipids with significant changes in cells showed a decrease in content after intervention, especially in LNCAP cells (see Figure 4B). Approximately 81.4% and 48.1% of the detected lipids were down-regulated by proxalutamide in LNCaP and 22RV1 cells, respectively. As the most detected lipids, more than half of TGs were down-regulated by proxalutamide, and the proportion in LNCaP cells was as high as 91.7% (see Figure 4C). These results suggest that proxalutamide could significantly change the lipid profile of the PCa cells and reduce the content of most lipids in the PCa cells, especially the TGs.

### 2.5. Proxalutamide Deceases Lipogenesis in PCa Cells

**Figure 5:**
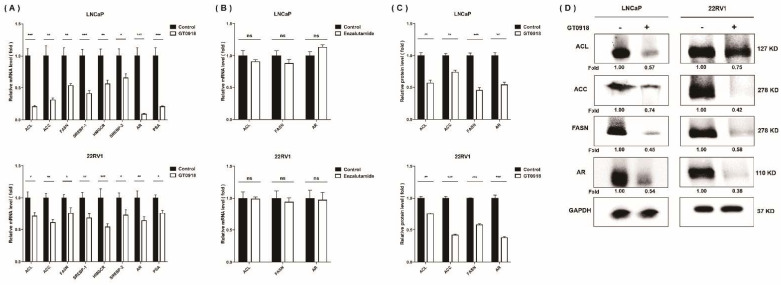
The effects of proxalutamide (GT0918) on AR expression and lipid de novo synthesis in PCa cells. (**A**) Proxalutamide significantly inhibited the mRNA expression of ATP citrate lyase (*ACL*), acetyl CoA carboxylase (*ACC*), fatty acid synthase (*FASN*), sterol regulatory element-binding protein-1/2 (*SREBP*-1/2), 3-hydroxy-3-methylglutaryl-coenzyme A reductase (*HMGCR*), prostate-specific antigen (*PSA*) and *AR* in LNCaP and 22RV1 cells. (**B**) Enzalutamide did not significantly affect the mRNA expression of *ACL*, *FASN* and *AR* in LNCaP and 22RV1 cells. The relative mRNA level was defined as 1.0 (fold) in the vehicle-treated (control) cells. Data were normalized by β-actin mRNA expression and shown as the mean ± SD (*n* = 5). ‘ns’ *p* > 0.05, * *p* < 0.05, ** *p* < 0.01, *** *p* < 0.001. (**C**,**D**) Proxalutamide significantly reduced the protein amounts of ACL, ACC, FASN and AR in LNCaP and 22RV1 cells. GAPDH was used as a loading control in Western blotting assay. The relative fold was defined as 1.00 in the vehicle-treated LNCaP or 22RV1 cells, respectively. Data were normalized by GAPDH protein expression and are shown as the mean ± SD (*n* = 3). ** *p* < 0.01, *** *p* < 0.001.

Compared with normal prostatic cells that mainly rely on dietary lipids to obtain fatty acids (FAs), the progression of PCa is characterized by an increased rate of de novo FA synthesis [42], independent of circulating lipid levels. The expression of enzymes and transcriptional regulators in the lipogenic pathway is significantly increased in PCa [43,44]. Meanwhile, it has been confirmed that a high level of cholesterol, a steroid lipid, in vivo could increase the risk of aggressive PCa [45]. To further evaluate the effect of proxalutamide on the abnormal lipid metabolism in PCa cells, we investigated the expression of genes linked to lipogenesis (see Figure 5). The results showed that in LNCaP and 22RV1 cells, proxalutamide significantly reduced the mRNA expression and protein levels of the key enzymes (ACL, ACC and FASN) and their upstream regulatory element (SREBP-1, sterol regulatory element binding protein-1) in de novo FA synthesis. Similar to FA synthesis, the mRNA expression of the key rate-limiting enzyme (*HMGCR*, 3-hydroxy-3-methylglutaryl-coenzyme A reductase) and its upstream regulatory element (*SREBP*-2) in cholesterol synthesis was also significantly inhibited by proxalutamide. These results also verified that proxalutamide can down-regulate the gene expression and protein expression of AR protein in AR-positive PCa cells. However, enzalutamide did not show any influence on the gene expression of *ACL*, *FASN* and *AR*. These results suggest that proxalutamide can down-regulate the rate of lipid synthesis and the protein level of AR in PCa cells.

## 3. Discussion

Prostate cancer (PCa) is a significant burden on men’s health. It is among the most frequently diagnosed malignancies in men in developed countries, and its incidence in developing countries has also increased significantly in the past decade [46]. Androgen deprivation therapy (ADT) has been a mainstay of treatment for PCa since the pivotal study of Huggins and Hodges in 1941 [47]. However, the cancer progresses, despite the low levels of circulating androgens that result from ADT treatment through surgical or medical castration. Androgen receptor (AR) is a nuclear hormone receptor that is activated in response to the binding of androgens, and its abnormality has been proven to be primarily responsible for the development, growth and progression of prostate tumors [48]. Despite the advancement of disease to castration-resistant prostate cancer (CRPC), the cancer cells remain dependent on the AR signaling pathway for growth [49]. However, even enzalutamide (Enz), a second-generation AR antagonist approved by the FDA for the treatment of CRPC, can only delay the progression of the disease within two years and subsequently develop resistance [50,51,52]. Therefore, the development of AR antagonists that efficiently and durably target this pathway are urgently needed, especially when CRPC patients develop resistance to Enz.

Proxalutamide, as a potential third-generation AR antagonist with the same core structure as Enz, was developed through computer-aided protein crystal structure optimization design. Several studies have demonstrated that despite Enz treatment, AR is highly expressed and transcriptionally active in CRPC due to the amplification of the acquired AR enhancer located at the AR 650 kb centromere [53,54]. Therefore, the down-regulation effects of proxalutamide on the gene and protein expression of AR would be beneficial for the inhibition of Enz-resistant CRPC tumors. Other previous studies have also confirmed this down-regulation effect of proxalutamide on AR protein [15,16]. Furthermore, the gene expression level of AR-V7 in 22RV1 cells was also significantly reduced after proxalutamide treatment (see Supplementary Materials). AR-V7 is the main splice variant of the full-length AR (wild-type AR) that encodes functional proteins [55]. Elevated levels of AR-V7 have been detected in tumor specimens and circulating tumor cells from patients with CRPC [56]. Moreover, resistance to the potent second-generation anti-androgens, e.g., ENZ and abiraterone acetate, has been attributed to the overexpression of AR-V7 [57,58]. However, after enzalutamide treatment, the gene expression of both wild-type AR and AR mutants (AR-V7) did not change significantly. This indicates that proxalutamide may be effective for patients who are resistant to ENZ.

Information on the metabolic mechanisms of the resistance mechanisms in CRPC is emerging. It has been found that a remarkable up-regulation of HMGCR, a key enzyme in cholesterol synthesis, mediates the Enz resistance of CRPC cells [59]. More studies suggest that the inhibition of de novo lipogenesis may be a promising method for overcoming AR-targeted therapy resistance in PCa [60,61]. However, this study found that proxalutamide can significantly inhibit the expression of key enzymes and transcription factors that regulate fatty acid synthesis and cholesterol synthesis in PCa cells. Therefore, it is possible for proxalutamide to retain efficacy against CRPC that has developed resistance to current AR-blockage treatments. In other words, the dual intervention of proxalutamide in the AR axis (e.g., AR expression and AR signal transcription [16]) and endogenous metabolism (e.g., lipogenesis and nucleotide synthesis [18]) will enable it to have a longer clinical service lifespan, from PCa to CRPC and drug-resistant CRPC. Interestingly, in our comparative studies, Enz was not found to have an effect on AR expression or endogenous metabolism in PCa cells. This may be a reason for the superiority of proxalutamide compared to Enz in inhibiting the proliferation and migration of PCa cells.

We further investigated the relationship between the above two anti-tumor mechanisms of proxalutamide. Proxalutamide was originally designed as an AR antagonist. However, it has been found that AR can also regulate the central metabolism and biosynthesis of PCa [62]. Therefore, we investigated whether the effect of proxalutamide on the metabolism will disappear after AR silencing. As shown in the Supplementary Materials, the silencing efficiency of the AR gene in LNCaP cells was above 80% under our established experimental conditions. However, the absence of AR did not affect the expression levels of the ACL, FASN or SREBP-1 genes in LNCaP cells. In addition, proxalutamide still significantly inhibited the gene expression of ACL, FASN and SREBP-1 in LNCaP cells after AR silencing. These results suggest that, as a newly developed AR antagonist, the inhibitory effect of proxalutamide on the de novo synthesis of fatty acids may not depend on its effect on the AR axis. Metabolic reprogramming may be a new independent mechanism for proxalutamide to enhance its efficacy. In general, this manuscript first confirmed that proxalutamide can significantly inhibit the proliferation and migration of PCa cells, and its effect is far superior to Enz. In addition, proxalutamide could induce caspase-dependent apoptosis of PCa cells. Subsequently, based on our previous research basis, we further investigated metabolic changes in PCa cells after proxalutamide intervention, focusing on abnormal lipid metabolism. The results showed that proxalutamide could significantly diminish the levels of lipid droplets in PCa cells, change the lipid profile of PCa cells and reduce the content of most lipids (especially triglycerides) in PCa cells. More studies have found that proxalutamide had a significant down-regulation effect on the gene and protein expression of key enzymes and transcription factors involved in the lipogenesis of PCa cells, and this effect does not depend on its ability to down-regulate AR expression. Based on in vitro cell models, we obtained many positive results, but whether these phenomena will change in vivo is still unknown. In the future, we will further verify the effect of proxalutamide on the endogenous lipid metabolism of PCa tumors in tumor-bearing mouse models, and the correlation between this effect and its effect on the AR signaling pathway.

## 4. Materials and Methods

### 4.1. Chemicals and Reagents

Proxalutamide (purity >99%) was supplied by Suzhou Kintor Pharmaceutical, Inc. (Suzhou, Jiangsu, China), and the compound code is GT0918. The corresponding carbon spectra, hydrogen spectra and HPLC spectra of proxalutamide are provided in the Supplementary Materials. Enzalutamide was purchased from Selleck Chemicals, Inc. (Houston, TX, USA). 14:0 Lyso PE was used as an internal standard (IS) in lipidomic profiling and was purchased from Sigma-Aldrich (St. Louis, MO, USA). Ultrapure water was prepared by a Milli-Q purification system (Millipore, Bedford, MA, USA). HPLC-grade acetonitrile and isopropanol were purchased from Merck (Darmstadt, Germany). Analytical-grade dimethyl sulfoxide (DMSO), ammonium acetate, acetic acid and methyl tert-butyl ether were purchased from Sigma-Aldrich (St. Louis, MO, USA). RPMI 1640 medium, fetal bovine serum (FBS) and penicillin–streptomycin (10,000 U/mL) were purchased from Gibco BRL (Grand Island, NY, USA). The Annexin V-FITC/PI Apoptosis Detection Kit was purchased from Vazyme Biotech Co., Ltd. (Nanjing, Jiangsu, China). BODIPY™ 558/568 C_12_ was purchased from Thermo Fisher Scientific (Waltham, MA, USA).

### 4.2. Cell Culture

Four human PCa cell lines, LNCaP, 22RV1, PC3 and DU145 were purchased from the Cell Bank of the Chinese Academy of Sciences (Shanghai, China). These cells were cultured in RPMI 1640 medium supplemented with 10% FBS and 1% penicillin–streptomycin at 37 °C with 5% CO_2_. LNCaP and 22RV1 are AR-positive PCa cells [63,64]. LNCaP cells retain the cytological characteristics of PCa tumors and their early differentiation function, which represents a significant feature of early androgen-dependent PCa [65]. The 22RV1 cell line was derived from a xenograft that was serially propagated in mice after castration-induced regression and relapse of a human prostatic carcinoma xenograft, and its growth is androgen-independent [66]. PC3 and DU145 are human metastatic PCa cells [67,68] without AR expression, and their growth is androgen-independent [69,70,71].

### 4.3. Analysis of PCa Cell Migration

Two metastatic human PCa cell lines (PC3 and DU145) were seeded into 6-well plates at a density of 2 × 10^5^ cells/well. A wound healing analysis was performed to measure cell motility. The cultured cell monolayers of two cell lines were wounded using a P200 micropipette tip in 6-well plates. The wounded monolayers were then incubated with proxalutamide (40 μmol/L) or vehicle (0.1% DMSO) in the absence of FBS for 24 h or 48 h. The scratch images were captured using a Leica inverted fluorescence microscope (Leica Microsystems, Heerbrugg, Switzerland) at 0 h, 24 h and 48 h. Then, the images were introduced into Image J software (National Institutes of Health, Bethesda, MD, USA) to calculate the wound area.

### 4.4. Analysis of PCa Cell Apoptosis

Four human PCa cell lines (LNCaP, 22RV1, PC3 and DU145) were seeded into 6-well plates at a density of 2 × 10^5^ cells/well. Then, they were treated with proxalutamide (40 μmol/L) or vehicle (0.1% DMSO) for 24 h. The treated cells were then collected and stained using the Annexin V-FITC/PI Apoptosis Detection Kit according to the manufacturer’s instructions. The apoptotic cells (%) were analyzed and calculated by flow cytometry (BD Bioscience, NJ, USA) and BD Accuri C6 software.

### 4.5. Quantitative Reverse Transcription-Polymerase Chain Reaction (RT-qPCR)

The AR-positive PCa cell lines (LNCaP and 22RV1) were seeded into 6-well plates at a density of 2 × 10^5^ cells/well, and then AR antagonist (proxalutamide or enzalutamide) or vehicle (0.1% DMSO) was used to treat the cells. Total RNA was extracted from these treated cells using a High Pure RNA Isolation Kit (RNAiso Plus, Takara, Japan), and then reversely converted into cDNA using a PrimeScript™ RT Reagent Kit (Takara Bio, Japan). The qPCR analysis was conducted using SYBR Premix Ex Taq™ (Takara Bio, Japan) and a CFX96 real-time PCR detection system (Bio-Rad, CA, USA). The gene-specific primers (shown in Supplementary Table S1) were synthesized by Invitrogen (Carlsbad, CA, USA). The cycling conditions of the qPCR program were as follows: 95 °C for 1 min, followed by 40 cycles at 95 °C for 15 s, 60 °C for 30 s and 72 °C for 30 s. Three replicates were prepared for each measurement. Melting curve analysis was performed to verify the specificity of real-time PCR products. Relative quantification was performed using the 2^−∆∆Ct^ method, and values were normalized to the reference gene β-actin.

### 4.6. Western Blotting

The AR-positive PCa cell lines (LNCaP and 22RV1) were seeded into cell culture dishes (Φ100 mm) at a density of 1 × 10^7^ cells/dish, and then proxalutamide (40 μmol/L) or vehicle (0.1% DMSO) was used to treat the cells. Extraction solution containing protease inhibitor and phosphatase inhibitor (Beyotime Biotechnology, China) was used to extract total protein samples from the treated cells. A Pierce™ BCA Protein Assay kit (Thermo Fisher Scientific, Waltham, MA, USA) was utilized to determine the protein concentration of the extracted sample. Equal amounts of protein samples were loaded into SDS-PAGE gels for electrophoresis, and then proteins were transferred onto polyvinylidene difluoride (PVDF) membranes. The blotted membranes were then blocked using 5% non-fat milk, followed by incubation with primary antibodies at 4 °C overnight. Subsequently, the secondary antibody was added and incubated at 37 °C for 1h. Primary antibodies used in this study were as follows: anti-AR (Abcam, Cambridge, MA, USA), anti-FASN/anti-ACC/anti-ACLY/anti-SREBP-1 (Cell Signaling Technology, Danvers, MA, USA) and anti-GAPDH (Bioworld Technology, Nanjing, Jiangsu, China). The secondary antibody was purchased from Thermo Fisher Scientific (Waltham, MA, USA). The protein signals were visualized using a BeyoECL Plus kit (Beyotime, China) and an imaging system (ChemiDoc MP System, Bio-Rad, CA, USA). Image J software was utilized to quantify specific protein bands.

### 4.7. Detection of LDs by Fluorescent Microscopy

The AR-positive PCa cell lines (LNCaP and 22RV1) were seeded into 6-well plates at a density of 2 × 10^5^ cells/well, and then AR antagonist (proxalutamide or enzalutamide) or vehicle (0.1% DMSO) was used to treat the cells. To visualize the LD formation in PCa cells, the treated cells were incubated with RPMI 1640 medium containing BODIPY 558/568 C_12_ at 37 °C for 30 min to fluorescently label the synthesized LDs. Subsequently, the stained cells were fixed with 4% paraformaldehyde, and then the nuclei were stained with Hoechst dye. The images were acquired by a Lionheart FX automated microscope (BioTek, Winooski, VT, USA) and introduced into Image J software to quantify the content of the LDs.

### 4.8. Lipidomics Analysis

#### 4.8.1. Lipidomics Sample Preparation

LNCaP cells were seeded into a 6-well plate at a density of 2.0 × 10^5^ cells/well, and then, proxalutamide (10 μmol/L) or vehicle (0.1% DMSO) was used to treat the cells. After the treatment, the cells were washed twice with PBS, and then ultrapure water was added to repeatedly freeze and thaw the cells. After the cells were lysed, 50 μL of the cell lysate was mixed with 5 μL IS solution (containing LPE14:0 at a final concentration of 200 μg/mL), 150 μL methanol and 500 μL methyl tert-butyl ether. After thoroughly mixing, 125 μL of ultrapure water was added to the mixed solution, and then the sample was centrifuged at 18,000 rpm for 10 min at 4 °C. After centrifugation, the supernatant was collected and evaporated to dryness. The residue was redissolved in a mixed solvent (acetonitrile:isopropanol:water = 60:35:5, *v*/*v*/*v*). The dissolved sample was centrifuged again to obtain the supernatant for analysis.

#### 4.8.2. Lipidomics Sample Detection

LC–MS/MS determination was performed using the LC-20A system (Shimadzu, Kyoto, Japan) coupled with the Qtrap™ 5500 system (SCIEX, Framingham, MA, USA). Chromatographic separation was achieved on a Thermo Hypersil Gold (C18) column (I.D. 2.1 mm × 100 mm, 5.0 μm, USA) with a column temperature of 40 °C. The mobile phase consisted of solvent A (containing 40% acetonitrile, 60% water, 5 mmol/L ammonium acetate and 0.1% acetic acid) and solvent B (containing 10% acetonitrile, 90% methanol, 5 mmol/L ammonium acetate and 0.1% acetic acid). A gradient elution at a flow rate of 0.2 mL/min was carried out: 0–2 min (25% B), 2–5 min (25–60% B), 5–23 min (60–90% B), 23–23.2 min (90–99% B), 23.2–26 min (99% B), 26–26.5 min (99–25% B) and 26.5–30 min (25% B). The mass spectrometer was equipped with an electrospray ionization (ESI) source, and MS analysis was operated both in positive and negative ionization modes using multiple-reaction monitoring (MRM). The source parameters for ESI were set as follows: ion spray voltage, 4500 V(+)/−4500 V(−); ion source gas1, 50 psi; ion source gas2, 50 psi; curtain gas, 30 psi; temperature, 550 °C. In the positive ionization mode, 552 lipids were set to be detected, the Q1 detection range was 300.2~972.8 Da (*m*/*z*), and the collision voltage was 10~50 V. In the negative ionization mode, 637 lipids were set to be detected, the Q1 detection range was 255.1~939.6 Da (*m*/*z*), and the collision voltage was −60~−10 V. Data acquisition and peak area integration were performed by Analyst TF 1.7 and MultiQuant 2.0 software from SCIEX, respectively. The peak area was used to represent the abundance of the target lipid in the sample.

#### 4.8.3. Lipidomics Data Analysis

The raw data were the peak area of each compound normalized by the peak area of the internal standard and the protein concentration of each sample. All data were imported into the MetaboAnalyst 5.0 website (https://www.metaboanalyst.ca, accessed on 11 September 2020) for multivariate analysis to screen the discriminant lipids that showed significant changes between the treated and untreated groups. When the difference in the abundance of the detected lipid between the treated group and the untreated group was greater than 2 times or less than 0.5 times, and there was a statistical difference (*p* < 0.05, *t* test), the lipid was regarded as the discriminant lipid between groups.

### 4.9. Statistical Analysis

All data were analyzed in at least three individual experiments by using a two-tailed unpaired Student’s *t* test for the comparison of independent means. The results were expressed as the mean ± S.D. The statistical analysis was conducted using GraphPad Prism 7.0 software (Graph Pad, San Diego, CA, USA). *p* values of less than 0.05 were considered statistically significant: * *p* < 0.05, ** *p* < 0.01, *** *p* < 0.001, **** *p* < 0.0001.

## 5. Conclusions

Through this research, we discovered a new strategy for the efficacy of proxalutamide against PCa progression, which is to cooperatively block the AR signaling axis and endogenous lipogenesis. To date, this metabolic reprogramming effect of proxalutamide on PCa cells has not been found in other AR antagonists, and endogenous metabolic abnormalities have been proven to be among the main mechanisms of PCa resistance. Therefore, proxalutamide may become a novel potential solution to overcome AR-targeted therapy resistance in PCa. Proxalutamide will have a much longer clinical service life, from PCa to CRPC and drug-resistant CRPC.

## Supplementary Materials

The following are available online at https://www.mdpi.com/article/10.3390/ijms222413222/s1.
