# Supplementary material for: Novel Strategy of Proxalutamide for the Treatment of Prostate Cancer through Coordinated Blockade of Lipogenesis and Androgen Receptor Axis
Source: Int J Mol Sci. 2021 Dec 8;22(24):13222. doi: 10.3390/ijms222413222 (PMC8704202; doi:10.3390/ijms222413222)
Supplement: Supplementary file 1 [file ijms-22-13222-s001.zip › ijms-1418917-supplementary.pdf]

Supplementary data

1. RT-qPCR profiling

Supplementary Table S1. Sequences of specific primers for the genes studied in this study.

Gene	Forward Primer (*5′-3′ Sequence*)	Reverse Primer (*3′-5′ Sequence*)
TNF-α	CCTCTCTCTAATCAGCCCTCTG	GAGGACCTGGGAGTAGATGAG
Caspase-8	GTTGTGTGGGGTAATGACAATCT	TCAAAGGTCGTGGTCAAAGCC
Cytochrome-c	CTTTGGGCGGAAGACAGGTC	TTATTGGCGGCTGTGTAAGAG
Caspase-3	GAAATTGTGGAATTGATGCGTGA	CTACAACGATCCCCTCTGAAAAA
ACL	ATCGGTTCAAGTATGCTCGGG	GACCAAGTTTTCCACGACGTT
ACC	ATGTCTGGCTTGCACCTAGTA	CCCCAAAGCGAGTAACAAATTCT
FASN	ACAGCGGGGAATGGGTACT	GACTGGTACAACGAGCGGAT
SREBP-1	ACAGTGACTTCCCTGGCCTAT	GCATGGACGGGTACATCTTCAA
HMGCR	GTCATTCCAGCCAAGGTTGT	GGGACCACTTGCTTCCATTA
SREBP-2	CCCCTGACTTCCCTGCTGCA	GCGCGAGTGTGGCCGGATC
AR	GACGACCAGATGGCTGTCATT	GGGCGAAGTAGAGCATCCT
PSA	GTGTGTGGACCTCCATGTTATT	CCACTCACCTTTCCCCTCAAG
β-actin	CATGTACGTTGCTATCCAGGC	CTCCTTAATGTCACGCACGAT

2. AR gene silencing

For gene silencing, siRNA transfection was performed using the Lipofectamine® 3000 Transfection Kit (Invitrogen, CA, USA) following the manufacturer's protocol. LNCaP cells were plated in 6-well plates at a density of 2×105 cells/well. It was ensured that the cell density was 30%-50% during transfection. For the gene silencing of AR, the 5'-3' sequence of the siRNA was designed as GGAGCUCUCACAUGUGGAATT, and the 3′-5′ sequence was UUCCACAUGUGAGAGCUCCTT. The final concentration of siRNA for cell incubation was 20 nmol/L. After incubating with siRNA at 37℃ for 48 h, the cells were treated with proxalutamide (40 μmol/L) or vehicle (0.1% DMSO). Finally, the gene expression of AR, ACL, FASN and SREBP-1 in the tested LNCaP cells was detected through RT-qPCR analysis. As shown in Supplementary Figure 1, the silencing efficiency of the AR gene in LNCaP cells was above 80% under our established experimental conditions. However, the absence of AR did not affect the gene expression of ACL, FASN or SREBP-1 in LNCaP cells. In addition, proxalutamide still significantly inhibited the gene expression of ACL, FASN and SREBP-1 in LNCaP cells after AR silencing. These results suggest that, as a newly developed AR antagonist, the inhibitory effect of proxalutamide on the de novo synthesis of fatty acids may not depend on its effect on the AR axis. The intervention effects on the endogenous metabolism of PCa tumors may be a new independent mechanism for proxalutamide to exert its efficacy.

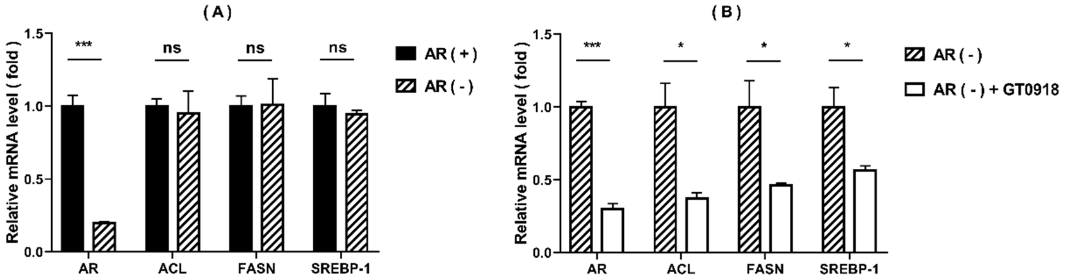

Supplementary Figure S1. The influence of AR silencing on the gene expression of AR, ACL, FASN and SREBP-1 in LNCaP cells (A) and the down-regulation effect of proxalutamide (GT0918) on the expression of these genes after AR silencing (B). AR(+) group represents normal LNCaP cells, AR(-) group represents LNCaP cells after AR

silencing, AR(-) + GT0918 group represents LNCaP cells in the AR(-) group after proxalutamide treatment. The relative mRNA level was shown as the mean ± SD (n = 3); 'ns' p>0.05, * p<0.05, *** p<0.001.

3. AR-V7 gene regulation

AR-V7 is the main splice variant of the full-length AR (AR-FL) that encodes a functional protein [1]. Elevated levels of AR-V7 have been detected in tumor specimens and circulating tumor cells from patients with castration-resistant prostate cancer (CRPC)[2] . Moreover, resistance to the potent second-generation anti-androgens, e.g., enzalutamide and abiraterone acetate, has been attributed to the overexpression of AR-V7 [3, 4]. LNCaP is an androgen-dependent PCa cell line that expresses only AR-FL. 22RV1 is an androgen-independent PCa cell line that expresses both AR-FL and AR-V7 [5]. In our study, it was found that proxalutamide can significantly down-regulate AR-FL gene expression in LNCaP and 22RV1 cells. Furthermore, we studied the changes in AR-V7 gene expression in 22RV1 cells. For the qPCR program, the 5'-3' sequence of AR-V7 was designed as CCATCTTGTCGTCTTCGGAAATGTTATGAAGC, and the 3′-5′ sequence was TTTGAATGAGGCAAGTCAGCCTTTCT. As shown in Supplementary Figure 2, the gene expression level of AR-V7 in 22RV1 cells was significantly reduced in proxalutamide-treated cells, while there was no significant change in enzalutamide-treated cells.

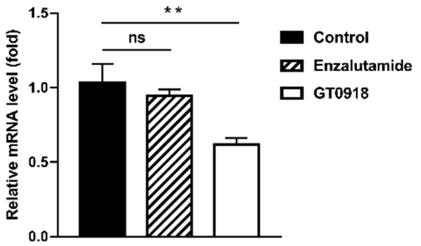

Supplementary Figure S2. The effects of proxalutamide (GT0918) and enzalutamide on AR-V7 expression in 22RV1 cells. The relative mRNA level was defined as 1.0 (fold) in the vehicle-treated (control) cells. Data were normalized by β-actin mRNA expression and are shown as the mean ± SD (n=5).

4. The purity of proxalutamide

```
=====================================================================
Sample Name    : LANB1616-19-3                       Seq. Line :    6
Acq. Operator  : PCX                                  Location : Vial 13
Method         : D:\HPCHEM\A040\METHODS\QCP2082.M    Inj Volume : 5 µl
```

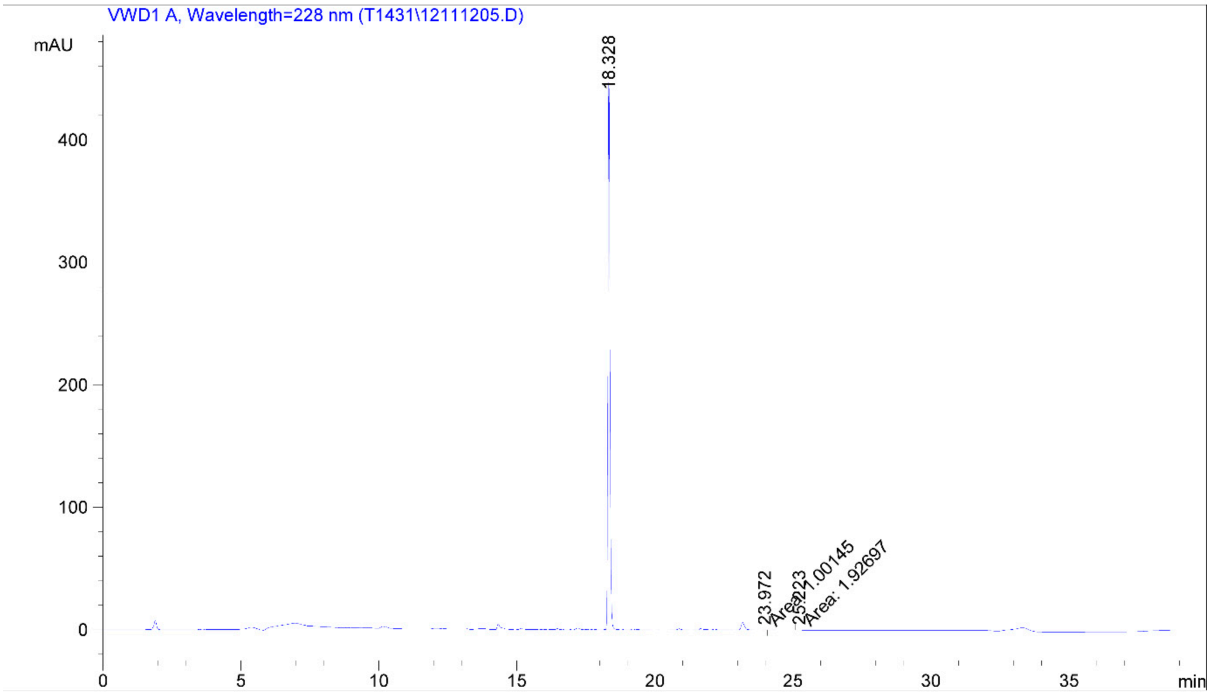


```
=====================================================================
                         Area Percent Report
=====================================================================

Sorted By             :      Signal
Multiplier            :      1.0000
Dilution              :      1.0000


Signal 1: VWD1 A, Wavelength=228 nm

Peak RetTime Type  Width     Area      Height      Area
  #   [min]        [min]  mAU    *s    [mAU    ]      %
----|-------|----|-------|----------|----------|--------|
   1  18.328 VV    0.0732 2211.19629  463.32169  99.8677
   2  23.972 MM    0.1170    1.00145 1.42677e-1   0.0452
   3  25.223 MM    0.1368    1.92697 2.34795e-1   0.0870

Totals :                  2214.12471  463.69916

 Results obtained with enhanced integrator!
=====================================================================
                          *** End of Report ***
```

Supplementary Figure S3. The HPLC spectra of proxalutamide used in this manuscript. The corresponding production batch number is LANB1616-19-3.

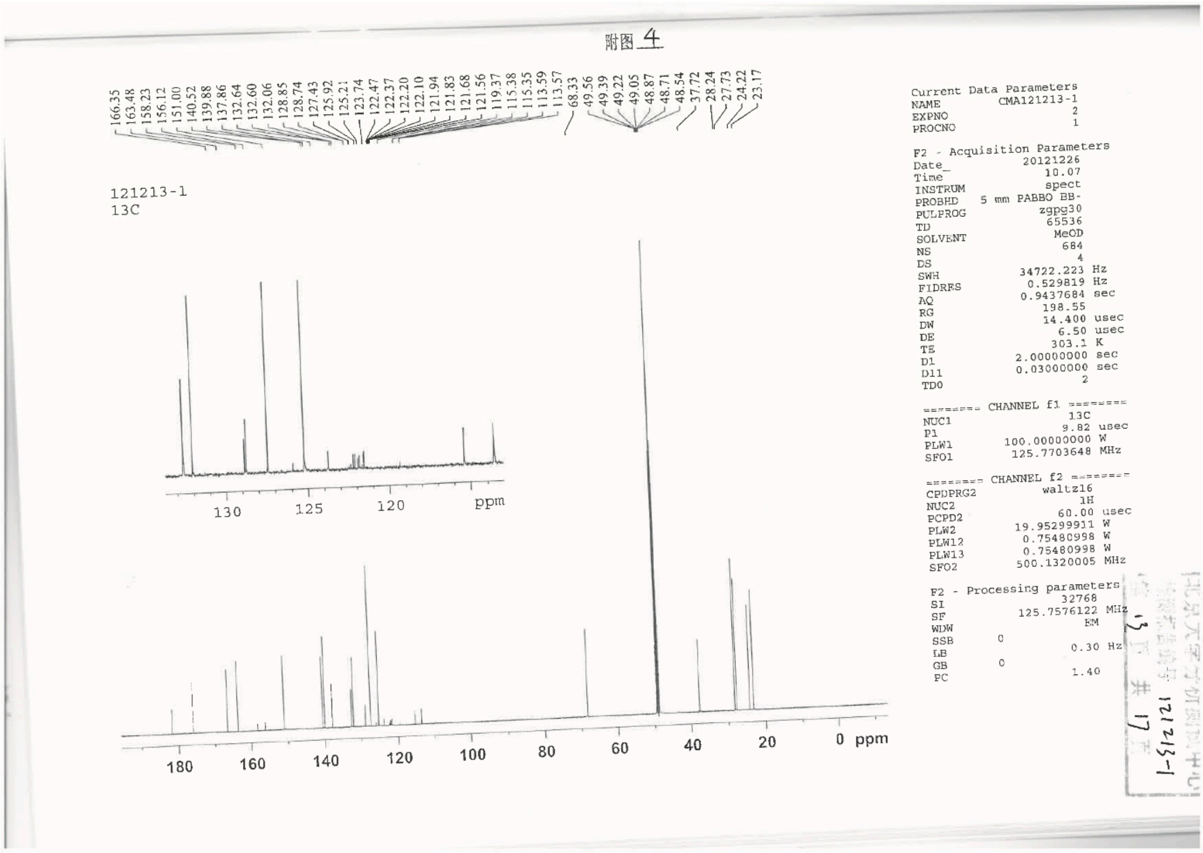

Supplementary Figure S4. The carbon spectra of proxalutamide used in this manuscript.

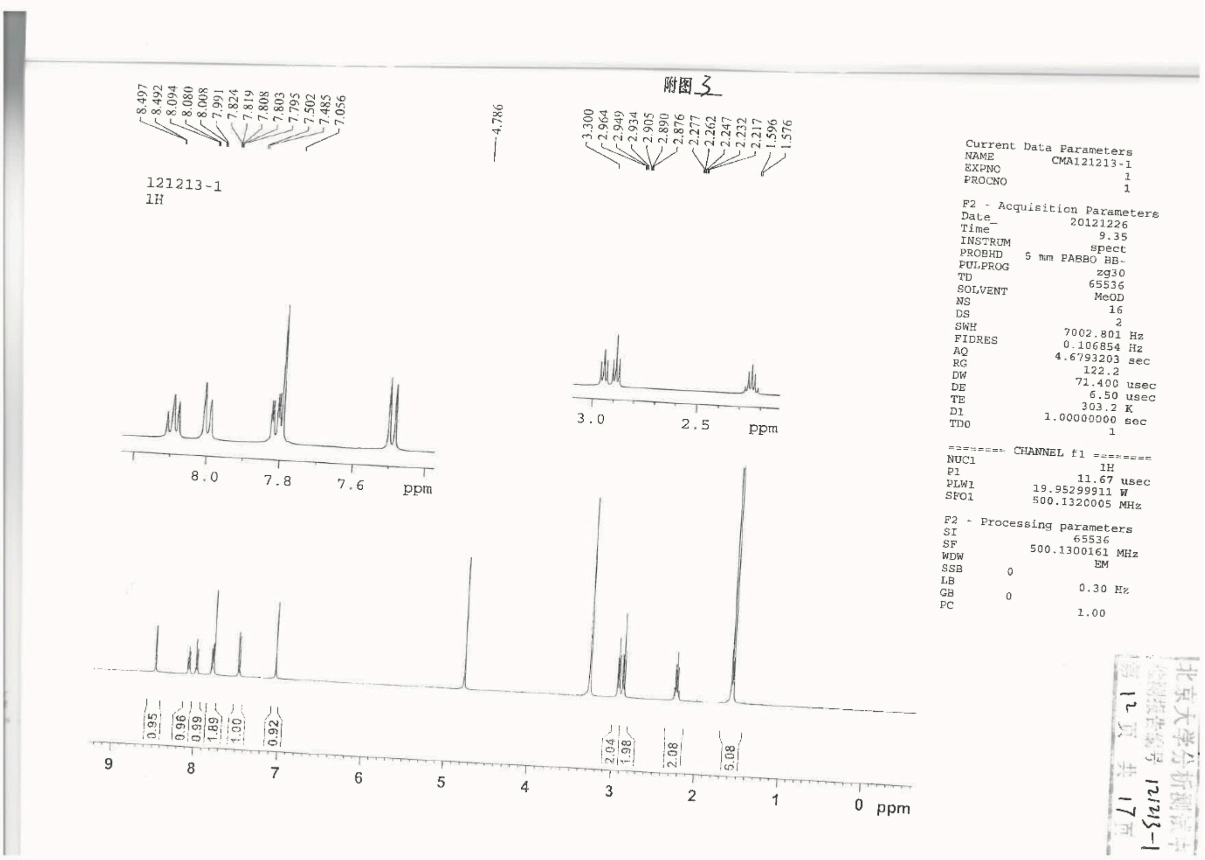

Supplementary Figure S5. The hydrogen spectra of proxalutamide used in this manuscript.

5. Drug toxicity and specificity of proxalutamide

Human colon cancer cells (HCT116) and normal human prostate epithelial cells (RWPE-1) were purchased from the Cell Bank of the Chinese Academy of Sciences (Shanghai, China), Human umbilical vein endothelial cells (HUVEC) were obtained from Promocell (Heidelberg, Germany). Each test cell line was seeded into a 96-well plate, and then were treated with proxalutamide at final concentrations of 1, 2, 5, 10, 20, 50, 100, and 200 μmol/L for up to 72 h. Cell viability was determined using a Cell Counting-Lite™ 2.0 luminescence kit method same to the reference [6]. In addition, HCT116 cells were seeded into 6-well plates, and then incubated with proxalutamide (40 μmol/L) or vehicle (0.1% DMSO) in the absence of FBS for 24h. The wound healing analysis was performed same to the method used for PCa cells in this study. As shown in Supplementary Figure 6B, the half inhibitory concentration (IC50) of proxalutamide was greater than 200 μmol/L in the growth of the three new cell lines tested. Among them, AR is expressed on RWPE-1 cells and HUVEC cells [7] , so the inhibitory effect of proxalutamide on these two cell lines is slightly stronger than that of HCT116 which does not express AR. Here, we quoted the data we reported earlier [6] , which are shown in Supplementary Figure 6A. The IC50 of proxalutamide for the growth of AR-positive prostate cancer (PCa) cells (LNCaP and 22RV1) is between 6.90 and -32.07 μmol/L, which is much lower than that of normal human cells (about 10 times). Therefore, we believe that the effect of proxalutamide has a cancer specific. Moreover, it can be seen from the Supplementary Figure 7 that proxalutamide has no effect on the migration of HCT-116 cells. HCT-116 cells do not express AR, but we have found that proxalutamide can significantly inhibit the migration of AR-negative PCa cells (PC3 and DU145), indicating that the migration inhibition of proxalutamide is prostate cancer cell specific.

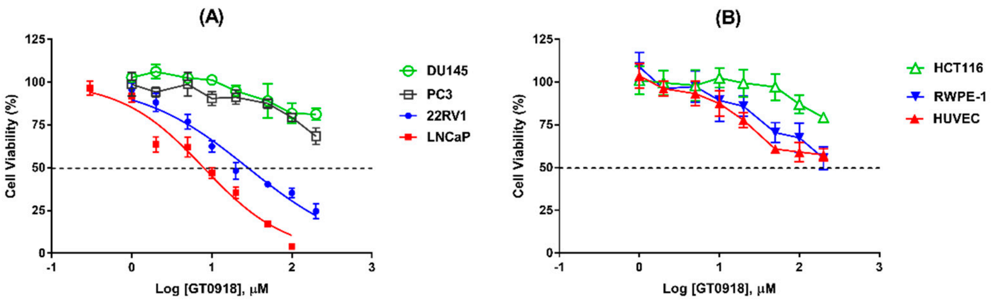

Supplementary Figure S6. The cell viability assay after the administration of a serial concentration (up to 200 μmol/L) of prxalutamide (GT0918). (A) Dose-response curves in prostate cancer cells, such as 22RV1 cells (blue), LNCaP cells (red), DU145 cells (green) and PC3 (black). (B) Dose-response curves in non-prostate cancer cells, such as HCT116 cells (human colon cancer cells / green), RWPE-1 cells (normal human prostate epithelial cells / blue) and HUVEC cells (human umbilical vein endothelial cells / red). The data in (A) comes from the research we reported earlier [6].

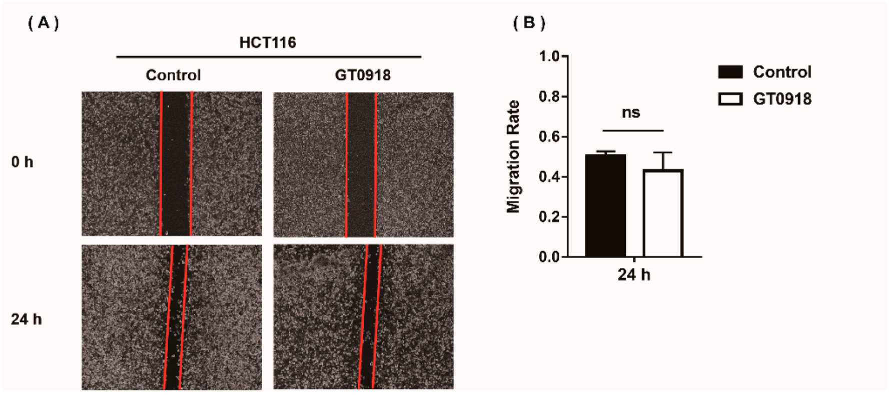

Supplementary Figure S7. Wound healing assay of HCT116 cells after exposure to proxalutamide (GT0918, 40 μmol/L) or control vehicle. Migration rate was defined as the ratio of the difference between the wound area at 0 h and 24 h after injury to the wound area at 0 h. Data represented the mean ± SD of three independent experiments, 'ns' p>0.05.

6. AR expression in normal and cancer cells

AR-positive PCa cell lines (LNCaP and 22RV1) and normal human prostate epithelial cells (RWPE-1) were seeded into 6-well plates at a density of 2×10^5 cells/well. The qPCR analysis of AR gene was performed same to the method used in this study. As shown in Supplementary Figure 8, the mRNA level of AR in AR-positive PCa cells is much higher than that in RWPE-1 cells, about ten thousand times higher.

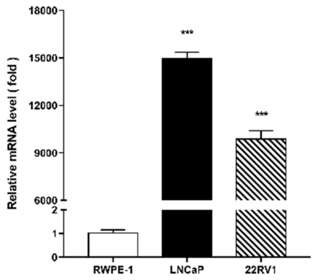

Supplementary Figure S8. Comparison of AR expression levels in normal human prostate epithelial cells (RWPE-1) and AR-positive prostate cancer cell lines (LNCaP and 22RV1). Data were normalized by β-actin mRNA expression and shown as the mean ± SD (n=3). The relative mRNA level was defined as 1.0 (fold) in the RWPE-1 cells. *** p<0.001.

References

1. Antonarakis, E.S., et al., *Androgen receptor variant-driven prostate cancer: clinical implications and therapeutic targeting.* Prostate Cancer Prostatic Dis, 2016. **19**(3): p. 231-41.
2. Zhang, X., et al., *Androgen receptor variants occur frequently in castration resistant prostate cancer metastases.* PLoS One, 2011. **6**(11): p. e27970.
3. Antonarakis, E.S., et al., *AR-V7 and resistance to enzalutamide and abiraterone in prostate cancer.* N Engl J Med, 2014. **371**(11): p. 1028-38.
4. Sarwar, M., et al., *Targeted suppression of AR-V7 using PIP5K1alpha inhibitor overcomes enzalutamide resistance in prostate cancer cells.* Oncotarget, 2016. **7**(39): p. 63065-63081.
5. Khurana, N., et al., *Multimodal actions of the phytochemical sulforaphane suppress both AR and AR-V7 in 22Rv1 cells: Advocating a potent pharmaceutical combination against castration-resistant prostate cancer.* Oncol Rep, 2017. **38**(5): p. 2774-2786.
6. Qu, F., et al., *Metabolomic profiling to evaluate the efficacy of proxalutamide, a novel androgen receptor antagonist, in prostate cancer cells.* Invest New Drugs, 2020. **38**(5): p. 1292-1302.
7. Liu, P.Y., et al., *Correlating androgen and estrogen steroid receptor expression with coronary calcification and atherosclerosis in men without known coronary artery disease.* J Clin Endocrinol Metab, 2005. **90**(2): p. 1041-6.
